# Effects of Electrical Stimulation of Acupoints on Xerostomia for Patients Who Undergo Hemodialysis

**DOI:** 10.3390/healthcare10030498

**Published:** 2022-03-09

**Authors:** Li-Yu Yang, Bih-O Lee, Kai-Ni Lee, Chien-An Chen

**Affiliations:** 1 College of Nursing, Kaohsiung Medical University, Kaohsiung 80708, Taiwan; a885019@kmu.edu.tw (L.-Y.Y.); biholee@kmu.edu.tw (B.-O.L.); 2 Department of Nephrology, Tainan Sinlau Hospital, Tainan 701002, Taiwan; 0430klc@gmail.com

**Keywords:** xerostomia, salivary flow rates, interdialytic weight gain, acupoints, hemodialysis

## Abstract

Xerostomia plays a major role in higher interdialytic weight gain (IDWG), which causes cardiovascular complications in patients who undergo hemodialysis. However, few studies have determined a method to manage xerostomia. This study determines the effect of transcutaneous electrical acupoint stimulation (TEAS) on hemodialysis patients with xerostomia and the percentage of IDWG. The study was a single-blind and quasi-experimental study. There are 75 participants: 37 in the TEAS group and 38 in the contrast group. The TEAS group used 250 µs and 50 Hz and the contrast group used 50 µs and 2 Hz three times a week for 3 weeks to stimulate ST 6 and TE17 acupoints. The salivary flow rates, dry mouth, and %IDWG were determined before, during and one week after the program. Compared with the contrast group, the TEAS group showed a significantly improved salivary flow rate (mL/min) (F (2, 123) = 15.28, *p* < 0.0001), and patients recovered their normal salivary flow rate. However, the results show that both groups showed significant improvement in dry mouth after treatment. The TEAS group demonstrated no effect in terms of %IDWG, as expected. The results show that a TEAS program is an effective means of symptom management for xerostomia patients who undergo hemodialysis. A TEAS program can be used to manage symptoms for xerostomia patients who undergo hemodialysis.

## 1. Introduction

Cardiovascular complications, such as high blood pressure and heart failure, are the most frequent causes of death for patients with end-stage renal disease (ESRD) [1]. The main cause of high blood pressure and heart failure is the retention of body fluid. Redundant fluid in the body is removed through regular dialysis, but patients who undergo hemodialysis are required to restrict liquid absorption, which is monitored using the principle that the total of interdialytic weight gain (IDWG) accounts for less than 5% of dry weight [2]. If the IDWG increases, patients are assumed to have consumed too much fluid. The results of many studies show that only 23% to 38% of patients comply with fluid restrictions [3,4,5].

There is some evidence that there is a positive correlation between xerostomia (hyposalivation) and IDWG [6,7,8]. A patient with more serious xerostomia experiences an increase in IDWG because of the increased fluid intake [9]. Clinically, dry mouth and xerostomia are common in patients who undergo hemodialysis, and dry mouth is the most common symptom for hemodialysis patients [10]. It has been shown that 35.9% to 66.1% of dialysis patients suffer from serious dry mouth and xerostomia [10,11]. Some studies also demonstrate that less saliva is produced by patients who undergo hemodialysis than by normal people [12,13,14]. A small amount of saliva (hyposalivary) may be a dangerous factor that results in xerostomia and IDWG for these patients.

Clinically, nurses advise patients to suck on ice cubes or chew gum to increase saliva secretion and relieve dry mouth. However, from the perspective of traditional Chinese medicine, cold foods have a negative effect on health, so most patients do not suck on ice cubes. Similarly, excessive gum chewing can lead to temporomandibular joint pain [15,16]. Previous studies showed that these two methods only provide short-term relief [15].

Acupoint stimulation is another method of increasing saliva secretion. The underlying mechanism that has been proposed for acupoint stimulation is that it affects the autonomic nerve system, causing the release of neuropeptides, which increase the production of saliva [17]. The results of studies on the use of acupuncture to treat xerostomia show that patients who receive treatment show significantly increased salivary flow rates versus baseline [18,19,20].

In a 14-week study, 38 patients were randomly allocated to receive either acupuncture or placebo acupuncture for two series of 12 treatments (each 20 min, twice a week). Each series lasted for 6 weeks, with a 2-week pause between the series. Saliva flow rates were measured at baseline, after the 12th of treatment (6 weeks); before a 13th treatment (8 weeks); after the completion of the acupuncture series (14 weeks); and then at 3, 6, and 12 months after the end of the acupuncture schedule. The improved salivary flow rates persisted during the observation period for both groups. The changes for the placebo group were somewhat smaller. The results showed that there were significant differences in the salivary flow rates only within each group and that there was no statistically significant differences between the groups [18]. A previous retrospective study by the authors that included 21 patients who received additional acupuncture treatment and 32 patients who chose not to continue acupuncture showed that after an initial course of 24 acupuncture treatments, patients who received additional acupuncture treatments have a consistently higher median saliva flow rates for 3 years after treatment compared with patients who choose not to continue acupuncture [19].The results of the two studies showed that the increased production of saliva persists during long-term follow-up [18,19]

Another systematic review study included 8 studies, involving 725 radiation-induced xerostomia patients. Sample sizes ranged from 12 to 339 patients. The results of this study showed that there is no significant difference between groups that receive acupuncture and placebo acupuncture in terms of the saliva flow rate, but acupuncture better resolved the symptoms of dry mouth [20]. 

These studies used acupoints to increase saliva flow rates. These acupoints were mainly in the regions of the parotid, submandivular, and labial glands, including ST 3; ST 6; ST 5; DU 20; ST 7; SI 17; LI 18; and the distal points, P 6, H 7, ST36, LIV 3, LI 11, LI 10, LI 4, SI 3, SP 8, SP 3, SP 6, KI 7, KI 3, and KI 5. Each patient received treatment between 12 times and 30 times, and each treatment lasted approximately 20–30 min [18,19,20].

Acupuncture cannot be practiced by unlicensed practitioners. Transcutaneous electrical nerve stimulation (TENS) is another method used to stimulate acupoints. This method uses skin electrodes to apply electrical stimulation to acupoints and is known as transcutaneous electrical acupoint stimulation (TEAS) [21]. TENS increases the saliva flow rate in healthy adults, in patients with radiation-induced xerostomia [22,23,24], and in patients who undergo hemodialysis [25]. The settings for the TENS unit are as follows: the pulse rate is 50 Hz, the pulse duration is 250 µs, and the unit is in normal mode. The effectiveness of TEAS in managing xerostomia and improving IDWG has rarely been determined so this study uses TEAS to reduce dry mouth and IDWG in xerostomia patients who undergo hemodialysis.

## 2. Materials and Methods

### 2.1. Design

A single blind, quasi-experimental study design was used. Measurements were taken at baseline, immediately after completion of the 3-week program and one week after the last session.

### 2.2. Participants and Setting

This study used subjects from the hemodialysis centers in two hospitals in South Taiwan from March to August 2014. Patients who were diagnosed as end-stage renal disease and who had received hemodialysis three times a week for more than three months; with a saliva flow rate of less than 0.1 mL/min at baseline and who were aged 20 years or more were recruited for the study. The exclusion criteria included patients with pacemakers, chronic heart failure, or Sjogren’s syndrome and those who had an infection, injury, or ulcer around the target stimulation points.

A total of 80 hemodialysis patients in the hemodialysis center of two hospitals were screened and 5 were excluded: 3 had a salivary flow rate of more than 0.1 mL/min at baseline and 2 patients in the TEAS group quit the study due to health problems. Seventy-five patients finished the study, giving a completion rate of 93.8%. The subjects on weekly hemodialysis schedules were divided into TEAS and contrast groups by drawing lots. The TEAS group underwent dialysis on Monday, Wednesday, and Friday, and the contrast group was treated on Tuesday, Thursday, and Saturday. There were 37 patients in the TEAS group and 38 in the contrast group.

During the study period, patients did not note undesired side effects, such as pain in the musculature or anesthesia of the skin. he sample size required to determine the difference between the two groups in terms of salivary flow rates, xerostomia scores, and %IDWG was estimated. The distribution conformed to a normal curve and was normally distributed for a sample size of 30 [26].

### 2.3. The Intervention

**Figure 1:**
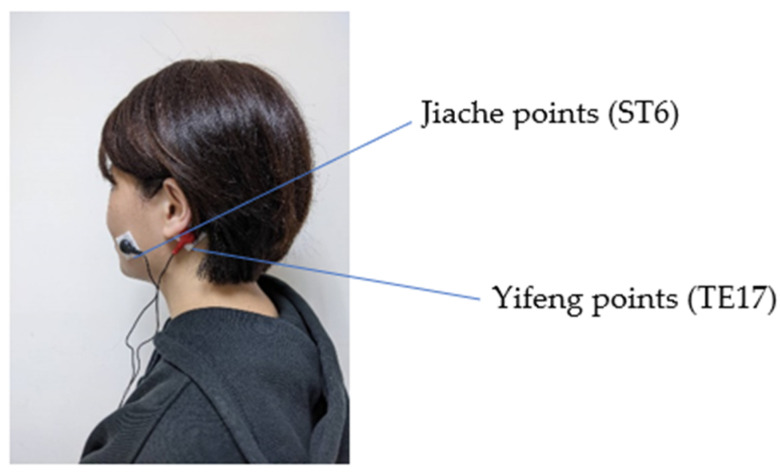
The TEAS used in the present study.

The intervention was performed three days a week for three weeks on patients who underwent dialysis. In a study by Yang et al. [25], the TEAS group was then given TENS at 50 Hz and a pulse duration of 250 µs to produce a tingling sensation three times a week for three weeks. Surface electrodes with dimensions of 2.5 × 2 cm^2^ were placed on acupuncture points ST6 and TE17, and stimulation lasted for 20 min, as shown in Figure 1. The contrast group received the same treatment as the TEAS group but the TENS used 50 µs and 2 Hz. Patients were less likely to perceive that they were in the contrast group in comparison with a study that uses no stimulation. All interventions were administered by an expert.

### 2.4. Instruments

Apart from the demographic information about the subjects, the instruments for this study include a Xerostomia Questionnaire (XQ) to assess the severity of dry mouth, oral cotton rolls, an electronic scale for salivary flow rates, % IDWG (IDWG divided by the dry weight), and ae transcutaneous electrical nerve stimulator. Demographic information about the subjects included gender; age; educational level; and relevant attributes of disease, including duration of HD, residual urine output, and changes in the severity of dry mouth over time.

### 2.5. Xerostomia Questionnaire (XQ)

There were eight items in the XQ, and a Likert 11-point scale from 0 to 10 was adopted for the scoring. The questions included four items about dryness while eating or chewing and four items about dryness while not eating or chewing. The sum scores were transformed linearly to produce a final summary score between 0 and 100 [27]. A higher score indicates more serious dry mouth. For the scale-based prediction, the Intra-class Correlation (ICC) was used to determine the stability of the items at intervals of 4 weeks. For this study, the reliability measure was 0.95 (*n* = 30).

### 2.6. Cotton Rolls and Electronic Balance

These two tools were used to measure the amount of saliva. Patients were prohibited from drinking water, taking food or smoking for one hour prior to the experiment in order to avoid triggering the secretion of saliva. Before the measurement, the patients swallowed the saliva in their mouth and cotton rolls were then inserted into the mouth for 5 min. The cotton rolls were removed and weighed using an electronic balance (METTLER B204S, Mettler Toledo, Zurich, Switzerland), which has a resolution of 0.0001 g. Each gram of saliva was converted into one millimeter of saliva. This value was divided by 5 min to determine the amount of saliva per minute for hemodialysis patients. A higher value indicated a larger quantity of saliva. To determine the reliability, a cotton roll that had absorbed water was weighed five times within 10 min. The coefficients of variation were all less than 5%, so cotton rolls were an accurate tool to measure saliva production. The device was calibrated before the experiment to decrease the error.

### 2.7. Transcutaneous Electrical Nerve Stimulator

The transcutaneous electrical nerve stimulator (D0207KL, Ching Ming, New Taipei, Taiwan) was a low-frequency electrical nerve stimulator, as specified by the Ministry of Health and Welfare of Taiwan (DOH-MD-No.000704). It consists of two double output lines and provides a current with an ae bandwidth of 50 µs to 250 µs and a frequency of 2 Hz to 150 Hz. Before the measurement, the device was placed at the correct location and was covered by a protective cover after calibration. The battery was adequately charged before each intervention measure in order to maintain the consistency and stability of the electrical nerve stimulation.

### 2.8. Accuracy in Locating the Stimulation Points

In the presence of two licensed traditional Chinese medicine doctors, two research fellows were 100% accurate in locating the Jiache points (ST 6) on both cheeks and the Yifeng points (TE 17) behind both ears to ensure accuracy in locating the stimulation points.

### 2.9. Data Collection

The participants for this study were collected with the approval of the Ethics Committee of the participating hospital (KMUHIRB-20120378). Prior to the TEAS intervention, the saliva flow rates, dry mouth severity, and %IDWG were measured as basic data on the third day of the weekly hemodialysis schedule. Electrode sheets (2.5 × 2 cm^2^) were then placed on the Jiache points (ST 6) and the Yifeng points (TE 17).

The TEAS group received transcutaneous electrical nerve stimulation for 250 µs at 50 Hz for 20 min three times a week for three weeks. Electrical nerve stimulation for 50 µs at 2 Hz (minimum stimulus intensity) was given to ten patients with hemodialysis to determine whether the intensity of the stimulation intensity affects salivary secretion. There is no statistically significant improvement in the salivary flow rate before and after the program (*p* = 0.1). The contrast group received the electrical nerve stimulation for 50 µs at 2 Hz using the same points so patients were less likely to perceive that they were in the contrast group in comparison with a study that uses no stimulation. Data for saliva flow rate, dry mouth severity, and %IDWG for each participant were collected to compare the difference before, right after completion of the 9 TEAS sessions, and one week after the last session.

### 2.10. Data Analysis

SPSS for Windows release 20.0 (IBM, New York, NY, USA) was used for the statistical analysis. In terms of the demographic information for the subjects, data about type and sequence were measured in the form of a frequency distribution and a percentage, and a descriptive statistical analysis of the mean and standard error was used for consecutive data. Before the intervention, a Chi-square test and an independent sample *t*-test were used to determine the basic attributes, salivary flow rate, dry mouth severity, and percentage of interdialytic weight gain (%IDWG) for subjects to determine whether the two groups were homogeneous. The differences between saliva flow rate, dry mouth severity, and the %IDWG for the TAES and contrast groups were measured using a two-way ANOVA and a mixed design. A *p*-value < 0.05 was considered statistically significant.

## 3. Results

### 3.1. Demographic and Clinical Information

In terms of gender, most of the subjects were female (54.7%). All the subjects were aged from 31 to 89, with an average age of 59.9 ± 13. Years of schooling ranged from 0 to 16 years, with an average of 6.8 ± 4.8 years. In terms of the duration of hemodialysis, the shortest period was 3 months and the longest was 283 months (23 years and 7 months), with an average period of 80.1 ± 62.4 months. The results show that there is no significant difference in age and years of schooling (*p* > 0.05), but there is a significant difference in the duration of hemodialysis (t = −3.28, *p* = 0.002) for the two groups (Table 1). The duration of hemodialysis is included in the co-variation adjustment in the statistical analysis.

For patients with past experience of dry mouth, forty-six of the patients (61.3%) reported no change in the severity of dry mouth over time, 22 (29.3%) reported the most severe dry mouth on the day following hemodialysis, and 7 (9.3%) reported the most severe dry mouth in the days after hemodialysis. Prior to commencing the TEAS treatment, patients reported a widely varying range of dry mouth severity from 6.3 to 78.1 scores, with an average score of 34.8 ± 18.0. The mean saliva flow rate at baseline was 0.05 ± 0.03 mL/min, with a range from 0.001 to 0.1 mL/min. The mean %IDWG was 4.1 ± 1.6, with a range from 1 to 8%. These results also show that the %IDWG for 64 patients (85.3%) is less than or equal 5%, with 11 patients (14.7%) having more than 5% of the dry weight. There is no significant difference in the distribution of relevant attributes of disease (Table 1).

At baseline, saliva flow rate is not significantly correlated with dry mouth severity (r = −0.2, *p* = 0.09) and with %IDWG (r = −0.06, *p* = 0.63) for the entire group of HD patients with xerostomia, and there is no relationship between dry mouth severity and %IDWG (r = 0.1, *p* = 0.41).

### 3.2. Effect of Electrical Stimulation of Acupoints

**Figure 2:**
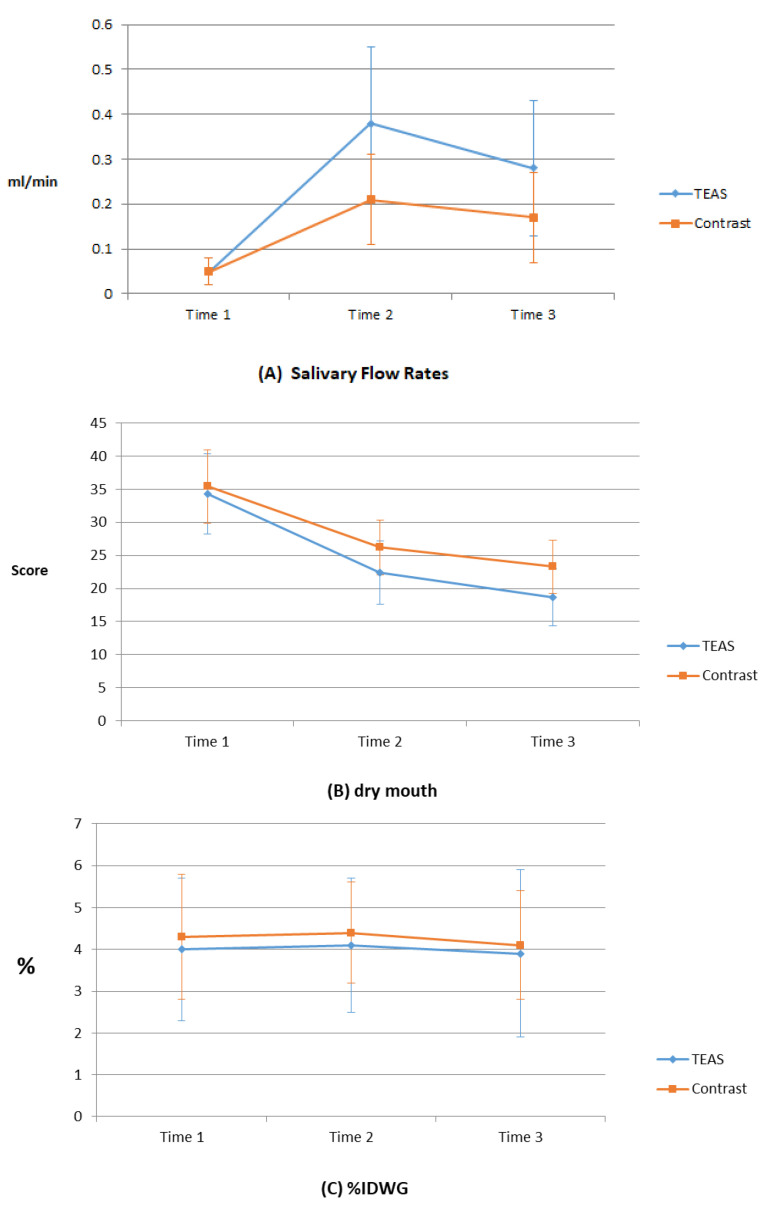
Effect of a TEAS program on saliva flow rate, dry mouth, and %IDWG: (**A**) saliva flow rates, (**B**) dry mouth, and (**C**) %IDWG. Time 1: before program; time 2: immediately post-program; and time 3: 1 week after program.

The effect of the TEAS treatments is shown in Table 2. The saliva flow rates for both groups increase over time. This result shows that salivary flow rates is an interaction (F (2, 123) = 15.28, *p* < 0.0001) between groups and times, with significant differences between both groups. The TEAS group shows a significant increase in saliva flow rate, compared with the contrast group (Figure 2A).

The severity of dry mouth for both groups decreases over the three time points but the result shows no significant difference between the groups in terms of dry mouth after TEAS treatments (F (2, 43.63) = 0.94, *p* = 0.39). However, the XQ scores for both groups decrease (*p* < 0.0001) during the intervention and 1 week after the end of the intervention (Figure 2B). 

The results also show that there is no significant difference (F (2, 9.99) = 0.44, *p* = 0.64) in the %IDWG between the two groups so there is no significant difference in weight gain between the two groups after TEAS treatments (Figure 2C). Most patients (85.3%) have an IDWG of less than or equal to 5% at baseline so generalized estimating equation (GEE) statistics is used to determine the IDWG changes at three time points for the two groups of patients whose IDWG is more than 5%. The parameter of the above statistical model was estimated with GEE to consider within-person variability. The GEE approach has been proposed as a non-parametric and appropriate method to conduct the repeated measurement analysis and to account for the correlated structure of disability data across repeated measurements. 

Eleven patients (14.7%) had more than 5% dry weight: six patients in the TEAS group and five patients in the contrast group at baseline. The results of GEE analysis show that there is a significant difference between the two groups over the three time points in terms of %IDWG (*p* = 0.016). For the TEAS group, there is a significant improvement in %IDWG (*p* = 0.006), but there is no significant improvement in %IDWG (*p* = 0.137) for the contrast group (Table 3).

## 4. Discussion

This study shows that, after the nine TEAS treatments, the amount of saliva that is produced demonstrates that stimulating the Jiache points (ST 6) and the Yifeng points (TE17) for 250 µs at 50 Hz increases the quantity of saliva for xerostomia patients that undergo hemodialysis. This result is also valid for the studies by Hargitai et al. [22], Lakshman et al. [23], and Yang et al. [25]. The saliva flow rate is also significantly increased for the contrast group (receiving low-parameter mode treatment), but saliva flow is greater in the TEAS group (high-parameter mode treatment) than the contrast group (F: 15.28, *p* < 0.0001). This shows that high-frequency and high-intensity electrical nerve stimulation is more effective at increasing the quantity of saliva. There is a significant clinical difference in the amount of saliva for each group before and after the interventional measure. However, low-frequency and low-intensity stimulation is shown to increase the saliva flow rate.

This study also shows that the average amount of saliva for xerostomia patients who undergo hemodialysis before TEAS is smaller than the definition of hyposalivation, which requires a rate of saliva flow before the stimulation of less than 0.1 mL/min. [28]. These patients have trouble chewing or swallowing solid food due to inadequate secretion of saliva and suffer discomfort in the mouth due to xerostomia.

The mean for the XQ scale before the interventional measure shows that dry mouth for xerostomia patients undergoing hemodialysis has a moderate to low level. The results show that a significantly low flow rate does not definitely cause severe dry mouth. This result was found by another study [29], which showed that the prevalence of xerostomia does not differ significantly between hemodialysis patients with reduced salivary secretions and individuals with un-stimulated saliva flow rates. Xerostomia can be caused by a low saliva flow rates and by changes in the composition of saliva [30].

Sensory adaptation may be another reason for this phenomenon. Subjects experience hyposalivation for a period of time so they subjectively perceive a reduced level of xerostomia, even though the stimulus continues. However, the results of this study show that the severity of dry mouth is not a predictor of saliva flow rate for xerostomia patients undergoing hemodialysis. Routine assessment of hemodialysis patients for salivary flow rate may prevent the development of severe xerostomia.

The average age of the subjects is less than 60 years. Age may contribute to an increase in the amount of saliva. Studies show that the salivary gland deteriorates with age [31] so TEAS is more effective for younger patients. This study uses a measurement that is taken 1 week after the end of treatment program and the secretion of saliva decreases slightly but is still more than 0.1 mL/min. 

The results of this study show that there is no significant association among the severity of dry mouth, the salivary flow rate, and %IDWG for xerostomia patients who undergo hemodialysis. TEAS has a significant effect on the quantity of saliva for the TEAS group but there is no significant difference in %IDWG and severity of dry mouth. Many studies show that the quantity of saliva and the severity of dry mouth are correlated with IDWG [32,33,34]. This study shows that there is no significant relationship between dry mouth, saliva flow rate, and increased %IDWG. There is no causal relationship between dry mouth and IDWG because patients with high IDWG who do not report xerostomia may drink a lot of water to reduce oral dryness or drink whenever they feel dry mouth. Patients may also feel very thirsty but refrain from drinking. This study demonstrates that TEAS treatment decreases %IDWG for more than 5% of IDWG patients who undergo hemodialysis with xerostomia. It is likely that the TEAS program reduces dry mouth, which leads to reduced IDWG. This result verifies the result that patients with IDWG  >  5% drink more than patients with IDWG  <  5% [35].

In summary, xerostomia is improved by the TEAS program, but it remains to be ascertained whether TEAS reduces %IDWG. Future randomized, controlled studies may show that this is accompanied by a reduction in %IDWG and dry mouth for patients with a higher IDWG who undergo hemodialysis.

### 4.1. Implication

The results show that the a 250 µs and 50 Hz TEAS program has an effect on xerostomia patients who undergo hemodialysis. The program is given three times a week for a total of 60 min for 3 weeks, and TEAS is then administered at intervals of one week to maintain a saliva flow rates of more than 0.3 mL/min. A tailed TEAS program can be used to manage symptoms for xerostomia patients who undergo hemodialysis in different settings or hospitals if appropriate.

### 4.2. Limitations

All samples were collected from only two hospitals, and the average age of the patients was less than 60 so it was impossible to determine whether the TEAS increases saliva in patients with hemodialysis who are older. More studies are required to increase generalization. Moreover, the both groups are not equal with regard to duration of HD treatment although the objective measures of salivary flow rate did not differ in the initial time point and, as such, may be subject to some bias. Additionally, studies show that the absorption of sodium contributes to an increase in IDWG in addition to dry mouth/xerostomia [36]. This study does not measure the absorption of sodium and fluid in the diet of the patients or the long-term effects so it is impossible to determine the length of time that is required to reduce water absorption when the saliva flow rate is increased and xerostomia is alleviated to reduce IDWG. Fluid absorption can be used as an effectiveness index for the interventional measure for future studies.

## 5. Conclusions

A TEAS program that stimulates acupoints ST6 and TE17 is a non-pharmacological therapy used to achieve a saliva flow rate of more than 0.3 mL/min. Xerostomia patients who undergo hemodialysis can achieve a reduction in the severity of xerostomia if they are subject to TEAS treatment. A TEAS program can be used to manage symptoms for xerostomia patients who undergo hemodialysis.

## Figures and Tables

**Table 1:** Demographic and clinical characteristics (*n* = 75).

Variables	TEAS (*n* = 37) *n*(%)/M (SD)	Contrast (*n* = 38) *n*(%)/M(SD)	Total (*n* = 75) *n*(%)/M(SD)	χ^2^/t *p*
Gender Female Male	18 (48.6) 19 (51.4)	23 (60.5) 15 (39.5)	41 (54.7) 34 (45.3)	0.30 ^b^
Age (years)	59.9 (13.0)	58.8 (11.5)	59.9 (13)	0.96 ^a^
Education (years)	6.9 (5.0)	6.6 (4.7)	6.8 (4.8)	0.83 ^a^
Duration of HD (months)	57.7 (54.3)	102 (62.6)	80.1 (62.4)	0.002 ^a^
Salivary flow rates (mL/min)	0.05 (0.03)	0.05 (0.03)	0.05(0.03)	0.69 ^a^
Dry mouth (score)	34.3 (18.9)	35.4(17.3)	34.8 (18.0)	0.80 ^a^
%IDWG	4.3 (1.9)	4.4 (1.5)	4.3 (1.7)	0.82 ^a^
Residual urine output 0 mL <100 mL ≧100 mL	12 (32.4) 17 (46.0) 8 (21.6)	17 (44.7) 11 (29.0) 10 (26.3)	29 (38.7) 28 (37.3) 18 (24.0)	0.31 ^b^
Most severe dry mouth no difference the day following HD the days after HD	24 (64.9) 9 (24.3) 4 (10.8)	22 (57.9) 13 (34.2) 3 (7.9)	46 (61.3) 22 (29.3) 7 (9.3)	0.62 ^b^

Notes: ^a^ *t*-test, ^b^ Pearson chi-square.

**Table 2:** Effect of electrical stimulation of acupoints for both groups (*n* = 75).

Variables	Time 1 M(SD)	Time 2 M(SD)	Time 3 M(SD)	F ^a^	Scheffe’ Post Hoc	F ^b^	F ^c^
Saliva flow rate (mL/min)						23.77 ***	15.28 ***
TEAS (*n* = 37)	0.05 (0.03)	0.38 (0.17)	0.28 (0.15)	38.39 ***	Time 2 > Time 1 ** Time 3 > Time 1 **		
Contrast (*n* = 38)	0.05 (0.03)	0.21 (0.10)	0.17 (0.10)	18.51 ***	Time 2 > Time 1 ** Time 3 > Time 1 **		
Dry mouth (score)						0.95	0.94
TEAS (*n* = 37)	34.3 (18.9)	22.4 (15.1)	18.7 (13.5)	22.64 ***			
Contrast (*n* = 38)	35.4 (17.3)	26.2 (12.9)	23.3 (12.4)	11.54 ***			
%IDWG (%)	4.0 (1.7)	4.1 (1.6)	3.9 (2.0)	0.07		0.94	0.44
TEAS (*n* = 37)							
Contrast (*n* = 38)	4.3 (1.5)	4.4 (1.2)	4.1 (1.3)	0.07			

Notes: After adjusting for covariates (hemodialysis duration), a two-way ANOVA, mixed design was used. *** *p* < 0.0001. ** *p* < 0.001. Time 1: before TEAS program, Time 2: immediately after completion of the TEAS program, Time 3: one week after completion of the TEAS program. ^a^ Time factor, ^b^ group, ^c^ time × group.

**Table 3:** Effect of electrical stimulation of acupoints for %IDWG (*n* = 11).

Group	Time 1 M(SD)	Time 2 M(SD)	Time 3 M(SD)	Wald χ^2^	*p*
Groups × Time				8.31	0.016
TEAS (*n* = 6)	6.6(0.4)	4.9(0.6)	5.1(1.2)	7.57	0.006
Contrast (*n* = 5)	6.7(0.9)	6.0(1.6)	5.9(1.8)	2.21	0.14

Notes: Generalized estimation equation analysis is used. Time 1: before TEAS program; time 2: immediately after completion of the TEAS program; time 3: one week after completion of the TEAS program.

## Data Availability

The data presented in this study are available upon request from the corresponding author. The data are not publicly available for ethical reasons.
